# Stimulus-Responsive Hydrogels for Targeted Cancer Therapy

**DOI:** 10.3390/gels10070440

**Published:** 2024-07-01

**Authors:** Raghu Solanki, Dhiraj Bhatia

**Affiliations:** Department of Biological Sciences and Engineering, Indian Institute of Technology Gandhinagar, Palaj 382355, Gujarat, India

**Keywords:** hydrogels, stimuli-responsive, drug delivery, cancer

## Abstract

Cancer is a highly heterogeneous disease and remains a global health challenge affecting millions of human lives worldwide. Despite advancements in conventional treatments like surgery, chemotherapy, and immunotherapy, the rise of multidrug resistance, tumor recurrence, and their severe side effects and the complex nature of the tumor microenvironment (TME) necessitates innovative therapeutic approaches. Recently, stimulus-responsive nanomedicines designed to target TME characteristics (e.g., pH alterations, redox conditions, enzyme secretion) have gained attention for their potential to enhance anticancer efficacy while minimizing the adverse effects of chemotherapeutics/bioactive compounds. Among the various nanocarriers, hydrogels are intriguing due to their high-water content, adjustable mechanical characteristics, and responsiveness to external and internal stimuli, making them promising candidates for cancer therapy. These properties make hydrogels an ideal nanocarrier for controlled drug release within the TME. This review comprehensively surveys the latest advancements in the area of stimulus-responsive hydrogels for cancer therapy, exploring various stimuli-responsive mechanisms, including biological (e.g., pH, redox), chemical (e.g., enzymes, glucose), and physical (e.g., temperature, light), as well as dual- or multi-stimuli responsiveness. Furthermore, this review addresses the current developments and challenges in hydrogels in cancer treatment. Our aim is to provide readers with a comprehensive understanding of stimulus-responsive hydrogels for cancer treatment, offering novel perspectives on their development for cancer therapy and other medical applications.

## 1. Introduction

Cancer is one of the most detrimental diseases, affecting millions of human lives globally and imposing a significant economic burden. As per the reports from the International Agency for Research on Cancer (IARC), there were about 20 million new cancer cases diagnosed, as well as 9.7 million cancer-related deaths in 2022 [1]. In 2040, about 28.4 million new cancer cases are expected [1,2]. Cancer is seriously affecting patients and their families and leads to high economic expenses including medical expenses and the loss of human capital due to early deaths [2]. Conventional cancer therapies, such as chemotherapy and radiotherapy, often suffer from limited efficacy, multidrug resistance, tumor recurrence, and severe side effects such as cardiotoxicity and nephrotoxicity due to their non-specific targeting of healthy tissues [3]. Recently, the emergence of cancer biomarkers in oncology has transformed cancer treatment, leading to significant advances in cancer therapies and improved patient prognoses [4]. Modern advances in interdisciplinary fields; the evolution of molecular imaging tools such as magnetic resonance imaging (MRI), computed tomography (CT), positron emission tomography (PET), single photon emission computed tomography (SPECT), and photoacoustic tomography (PAT) for cancer diagnostics [5,6]; developments in artificial intelligence (AI) in oncology [7]; significant advancements in single-cell multi-omics technology such as FIPRESCI [8]; and cancer vaccines [9] have revolutionized cancer therapy. However, these advances in cancer therapy are still insufficient to combat cancer completely. Hence, there is a pressing need to explore innovative approaches that can enable targeted drug delivery to cancer cells while minimizing systemic toxicity.

Nanotechnology represents a growing and promising field with diverse applications in drug delivery, bioimaging, and therapeutics [10]. Nano-drug delivery systems offer beneficial properties such as enhanced bioavailability, sustained drug release, stability, and improved aqueous solubility, thereby enhancing the anticancer activity of drugs [11]. Various nanocarriers, including hydrogels; polymeric, metallic, and lipid-based nanocarriers; micelles; dendrimers; and so on, have been utilized for the delivery of chemotherapeutic drugs/bioactive compounds [12].

Stimulus-responsive nanomedicine offers significant potential advantages in the realm of targeted therapy and personalized medicine [13]. By leveraging nanomaterials that respond to specific physiological stimuli—such as pH changes, temperature variations, or enzyme presence—these advanced therapeutic systems can deliver drugs with high precision directly to diseased tissues; minimizing off-target effects and reducing systemic toxicity. This targeted approach not only enhances the efficacy of treatments, particularly in cancer and inflammatory diseases, but also allows for controlled drug release, improving patient compliance and outcomes. Additionally, the adaptability of these nanomedicines can lead to real-time monitoring and responsive treatment adjustments, paving the way for more dynamic and effective healthcare solutions.

Hydrogels are three-dimensional (3D) structures of cross-linked polymers and holds great potential in drug delivery [14]. Their water-holding characteristics and structural similarity to biological tissues make them suitable carriers for drug delivery and tissue bioengineering applications. Hydrogels have modifiable durability, biocompatibility, and biodegradability as well as versatility in incorporating a wide range of therapeutic agents. Their adaptability for modification makes hydrogels a promising tool in improving the efficacy and safety of cancer treatments. Other advantages such as swelling behavior, porosity, mechanical properties, and responsiveness to various stimuli also make them ideal platforms for developing smart drug delivery systems. The TME has specific characteristics such as hypoxia, acidity, increased enzyme levels, and aberrant temperatures, all of which can be used to selectively induce drug release from hydrogels. In response to specific stimuli present in the TME, hydrogel-based drug delivery systems could be a promising approach to combat cancer.

**Figure 1:**
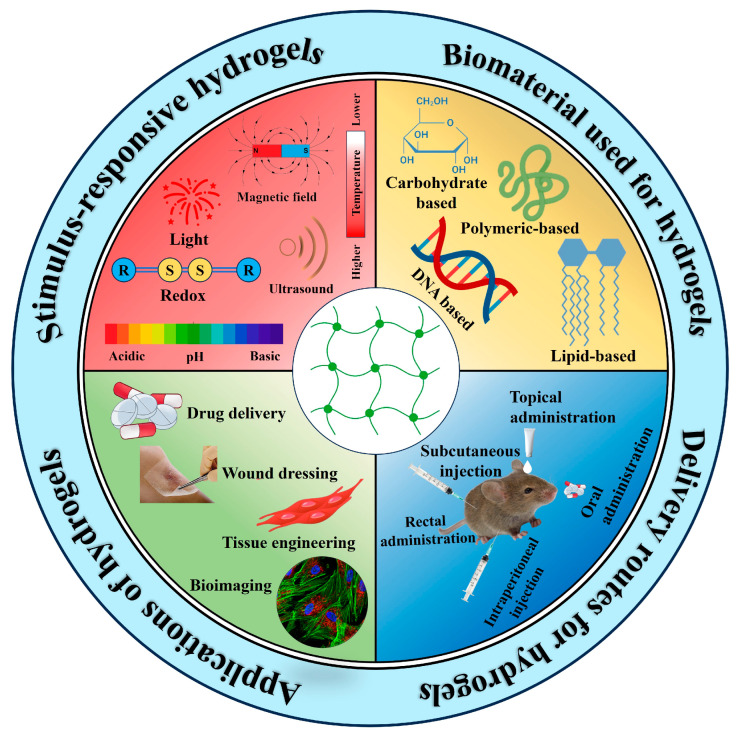
Stimulus-responsive hydrogels: synthesis materials, route for administration, and applications.

Targeted drug delivery through hydrogels is achieved via various stimuli such as pH, temperature, light, and so on [15]. Hydrogels can be synthesized using different biomaterials, including carbohydrates, proteins, lipids, or DNA/RNA-based nanomaterials, depending on the targeted cancer sites [16,17]. Hydrogels can be administered through various methods including injection, topical application, or implantation, depending on the therapeutic need. Hydrogels are widely used in biomedical applications such as drug delivery systems, wound dressings, and tissue engineering due to their biocompatibility and ability to mimic the TME (Figure 1).

We present here an in-depth overview of the current status of the research on stimulus-responsive hydrogels as a cancer therapy. We will explore the various types of biomaterials used in hydrogel fabrication, their responsiveness to different stimuli, and their applications in cancer targeting (Figure 1). Biological, chemical, and physical stimuli are thoroughly discussed with recent studies on hydrogels. By defining the fundamental concepts and focusing on current advances in the field, we are interested in shedding light on the potential of stimulus-responsive hydrogels to transform cancer treatment strategies.

## 2. Tumor Microenvironment (TME)

The scientific community has been unable to eliminate cancer despite many years of effort. One reason behind this is the complex nature of the TME. A tumor is a heterogeneous collection of invading cells rather than just a collection of cancer cells. This comprises the extracellular matrix (ECM), substances that are secreted, and resident host cells. In order to facilitate the growth and spread of tumors, cancer cells fundamentally alter the host tissues’ cellular, molecular, and physical makeup [18]. A developing TME is a dynamic and evolving entity. While the TME’s makeup varies depending on the kind of tumor, the immune cells, stromal cells, blood vessels, and extracellular matrix are its common components. The theory states that the “tumor microenvironment actively promotes the progression of cancer rather than merely acting as a silent bystander” [19]. The development of the TME is primarily linked to the uncontrolled growth of cancer cells and the expansion of defective blood vessels.

Recent research has revealed that key cellular metabolism pathways, which are controlled by internal genetic changes and TME-induced cell-extrinsic responses, are different in cancer cells from those in most normal tissue cells. These changes involve, but are not restricted to, altered signal transduction (e.g., hypoxia-inducible factor 1 (HIF-1), aerobic glycolysis, reduced oxidative phosphorylation) and biomolecule metabolism (e.g., glucose, lactate, glutathione). These factors lead to increased tumor proliferation, invasiveness, metastasis, and strong treatment resistance, enabling tumor cells to elude tumor-targeted therapies. They also promote vascular regeneration, nutrient uptake, adenosine triphosphate (ATP) generation, increased macromolecule biosynthesis, and elevated redox levels [20].

**Figure 2:**
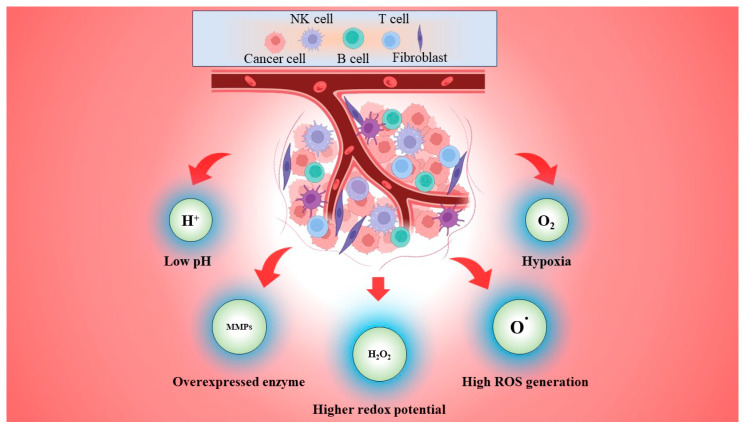
Schematic representation of tumor microenvironment (TME) and conditions prevailing in it, which are utilized as stimuli for targeted drug delivery to tumors.

Acidic pHs, hypoxia, increased levels of GSH, higher reactive oxygen species (ROS) generation, and overexpressed enzymes are key characteristics of the TME, which promote tumor angiogenesis and metastasis while also being responsible for therapeutic resistance and treatment failure (Figure 2) [21]. The design and development of TME-responsive intelligent drug delivery systems has gained interest in improving drug therapeutic effects owing to their potential to address some significant therapeutic issues, such as low therapeutic efficacy and serious side effects like cardiotoxicity, nephrotoxicity, and so on.

## 3. Hydrogels: An overview

**Figure 3:**
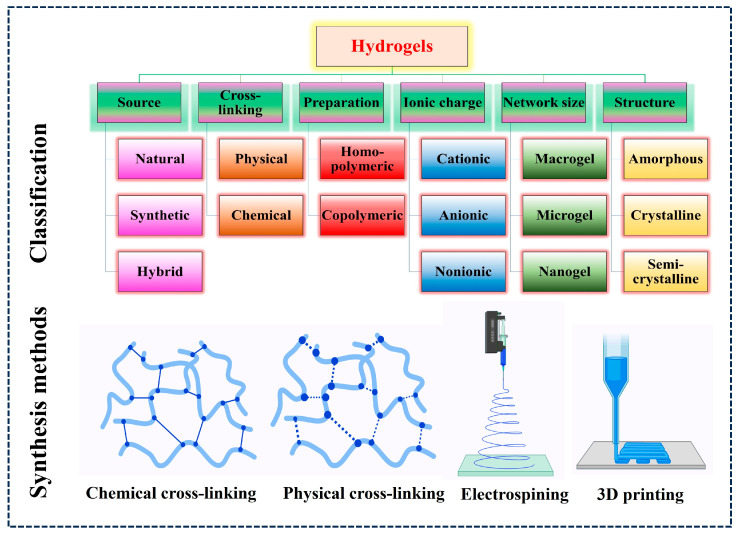
Classification and common synthesis methods for preparation of hydrogels.

In 1960s, Wichterle and Lim invented the word “hydrogel” to describe a 3D structure composed of hydrophilic polymers in biological contexts [22]. They named it poly (2-hydroxyethyl methacrylate) (pHEMA), and it was utilized in the contact lens industry [23]. pHEMA demonstrated how a hydrogel could absorb moisture and maintain its network structure. Hydrogels have emerged as fascinating drug delivery materials because of their distinct characteristics. One of their primary advantages is their high-water content, which replicates the natural environment of biological tissues, enhancing compatibility and decreasing discomfort during administration [24]. Furthermore, hydrogels have variable porosity and swelling behaviors, which allows for controlled drug release kinetics and maintains therapeutic concentrations over time [25]. Their soft and flexible structure allows for high entrapment of anticancer drugs including hydrophilic and hydrophobic drugs, while maintaining stability and bioactivity [26]. Furthermore, hydrogels have the benefit of being easily adaptable to achieve sustained release profiles for various pharmaceutical and medicinal applications. These promising characteristics make hydrogels an appealing candidate for precise and effective drug delivery systems. Hydrogels can be classified as homo or copolymeric based on their chemical composition; macro gels, microgels, or nanogels based on their network size; anionic, cationic, zwitterionic, or non-ionic based on their ion charge; and physical or chemical based on their cross-linking (Figure 3) [27,28].

Hydrogels can be synthesized using polymers through covalent or non-covalent bonding and classified into physically cross-linked, chemically cross-lined or hybrid cross-linking (Figure 3) [27]. Physical hydrogels are formed through interactions such as hydrogen bonding, Van der Waals interactions, ionic forces, polyelectrolyte complexation and hydrophobic forces which cause cross-linking among the polymer chains [29]. Due to the weak secondary connections between the polymer chains, physical hydrogels respond reversibly to environmental changes and are mechanically weak, disorganized, and fragile when subjected to external stimuli. In contrast, chemically cross-linked hydrogels are formed by covalent bonding between the polymer chains. Physically cross-lined hydrogels typically disintegrate in organic solvents and water (when heated) due to weak interactions, whereas chemical hydrogels do not dissolve in the surrounding medium and do not exhibit a reversible sol-gel transition. Chemical cross-linking agents such as formaldehyde, glutaraldehyde (GA), genipin, diglycidyl ether, and N, N′-methylenebisacrylamide stabilize the network by forming covalent bonds between small molecules and polymers through condensation or free radical mechanisms [30]. This process imparts excellent thermal, mechanical, chemical, and surface properties to the hydrogels, maintaining their network structure even when fully swollen. Additionally, advanced synthesis methods such as electrospinning and 3D printing (Figure 3) have been employed to create hydrogels with tailored structures and properties, enabling precise control over their architecture and functionality for various biomedical applications [31,32].

## 4. Stimulus-Responsive Hydrogels for Cancer Therapy

Stimulus-responsive hydrogels for cancer therapy represent a promising approach in drug delivery and treatment strategies. These hydrogels can be tailored to release therapeutic agents in response to specific stimuli such as pH, temperature, light, or enzymatic activity found in tumors, allowing for targeted and controlled drug delivery. Their ability to integrate with biological systems and respond dynamically to the TME makes them valuable tools for enhancing the efficacy and reducing the side effects of cancer treatments [33]. Stimulus-responsive hydrogels, a novel class of smart biomaterials, are engineered for drug delivery applications that utilize physical stimuli (such as temperature and light), chemical stimuli (such as pH and redox reactions), biochemical signals (such as glucose and enzymes), and can respond to dual or multiple stimuli.

**Figure 4:**
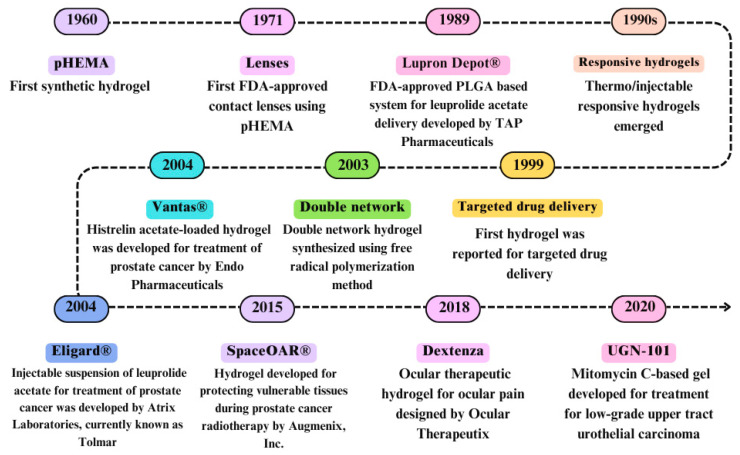
Some important developments in hydrogels as drug delivery systems.

These hydrogels exhibit responsiveness to stimuli, enabling controlled modulation of therapeutic agent release. This capability facilitates precise and controlled drug delivery strategies, thereby enhancing treatment efficacy and minimizing adverse effects in medical applications. Several hydrogel-based formulations have been developed over time for cancer treatment, prevention, and diagnosis with some undergoing clinical trials. More than 30 hydrogels have received FDA approval and are now used in clinical applications. Recently, UGN-101^®^ (Jelmyto™, formerly MitoGel, UroGen Pharma, Israel) [34] and Vantas^®^ (Endo Pharmaceuticals) [35] have been approved by the FDA for the treatment of cancers. UGN-101 is a temperature-sensitive, water-soluble, and novel polymer-based formulation of mitomycin C for the treatment of upper urothelial carcinomas [36]. The hydrogel composition is liquid at room temperature and gels at body temperature, conforming to each patient’s unique pelvicalyceal and ureteric anatomy. Vantas is a hydrogel depot made of polymers (hydroxyethyl methacrylate and hydroxypropyl) cross-linked with trimethylopropane trimethacrylate [37,38]. It releases histrelin acetate into the upper arm over 12 months following subcutaneous implementation, providing palliative treatment for advanced prostate cancer. Another FDA-approved gel-based product, ELIGARD^®^ (Atrix Laboratories, currently known as Tolmar) is an in situ polymeric gel used for the delivery of leuprolide acetate and has been approved for palliative therapy in advanced prostate cancer [38,39]. Utilizing a novel in situ polymeric gel extended delivery system, this formulation forms a solid implant upon injection. This allows for the controlled and sustained release of leuprolide acetate as the biodegradable gel. These are just some examples of hydrogels that are currently used in clinical practice (represented in Figure 4), but more research is ongoing, with many being tested in preclinical and clinical trials. In this review, we will discuss various stimulus-responsive hydrogels, providing recent examples from research studies.

In addition to significantly increasing the therapeutic efficacy of the loaded medications, responsive hydrogels can recognize tumor tissue and release drugs on demand based on specific conditions within the tumor microenvironment, thereby reducing harm to healthy tissues. These smart materials respond to multiple stimuli, such as changes in pH, temperature, and the presence of certain molecules. For instance, redox–pH hydrogels respond to altered glutathione in the cytoplasm of tumor cells, with the acidic TME triggering drug release, while thermo-pH-sensitive hydrogels respond to the elevated temperature and acidic environment of tumor cells. This targeted approach ensures effective drug delivery and controlled in situ release, enhancing treatment outcomes and minimizing side effects on normal tissues. The detailed mechanism of hydrogels in response to different stimuli are discussed in depth in their respective sections.

### 4.1. Chemical Stimuli-Responsive Hydrogels

Chemical response-mediated hydrogels dynamically change their structure or properties in response to specific chemical signals, offering tailored solutions for drug delivery and tissue engineering [40]. pH-responsive hydrogels undergo swelling or contraction in response to changes in environmental acidity or alkalinity, making them ideal for targeted drug release in acidic tumor microenvironments [41]. Similar to this, redox-responsive hydrogels can be made to change reversibly in response to changes in the redox state of their environment. This allows for controlled release in environments that are either reducing or oxidative [42]. We discuss pH- and redox-responsive hydrogels below, along with several research studies that have utilized these stimuli for drug delivery.

#### 4.1.1. pH-Responsive Hydrogels

Hydrogels that respond to pH fluctuations are an exceptional category of biomaterials designed to exhibit reversible changes [43]. These hydrogels contain pH-sensitive functional groups, such as acidic or basic residues, within their polymer networks, enabling them to swell or contract in acidic or alkaline environments. This pH-triggered swelling behavior can be harnessed for targeted drug delivery, where the hydrogel acts as a carrier for therapeutic agents, releasing them selectively in response to specific pH conditions characteristic of diseased tissues or cellular compartments [44]. Their tunable responsiveness to pH gradients renders them invaluable tools in biomedicine, offering tailored solutions for controlled release, tissue engineering, and diagnostic applications, with the potential to revolutionize drug delivery strategies and improve healthcare outcomes [45]. pH-sensitive hydrogels can deliver oral drugs to sites in the gastrointestinal (GI) tract due to pH variations [43].

Hydrogels act as cargo for therapeutic drugs, gradually releasing them through interactions with charged ions that modulate the release rate [46]. Polymers like PEG, PAA, pluronics, and chitosan are preferred for targeted drug delivery due to their pH-responsive nature, facilitated by protonable cationic (NH_2_) or deprotonable anionic (-COOH) groups in the hydrogel matrix. Adjusting pH levels effectively controls the swelling capacity of pH-responsive hydrogels. The ionization of hydrogels is altered with pH changes, influencing the swelling behavior of cross-linked polyelectrolytes [47]. Variations in pH, ionic strength, and electrostatic repulsion significantly affect these characteristics, making pH-sensitive hydrogels suitable for targeted oral drug-release formulations, leveraging pH differences in body regions like the colon (pH > 7) and stomach (pH < 3) [48]. Cationic hydrogels, such as those composed of polycationic polymers, exhibit maximal drug release at low pH levels, highlighting their utility in stomach-targeted drug delivery systems [49].

**Figure 5:**
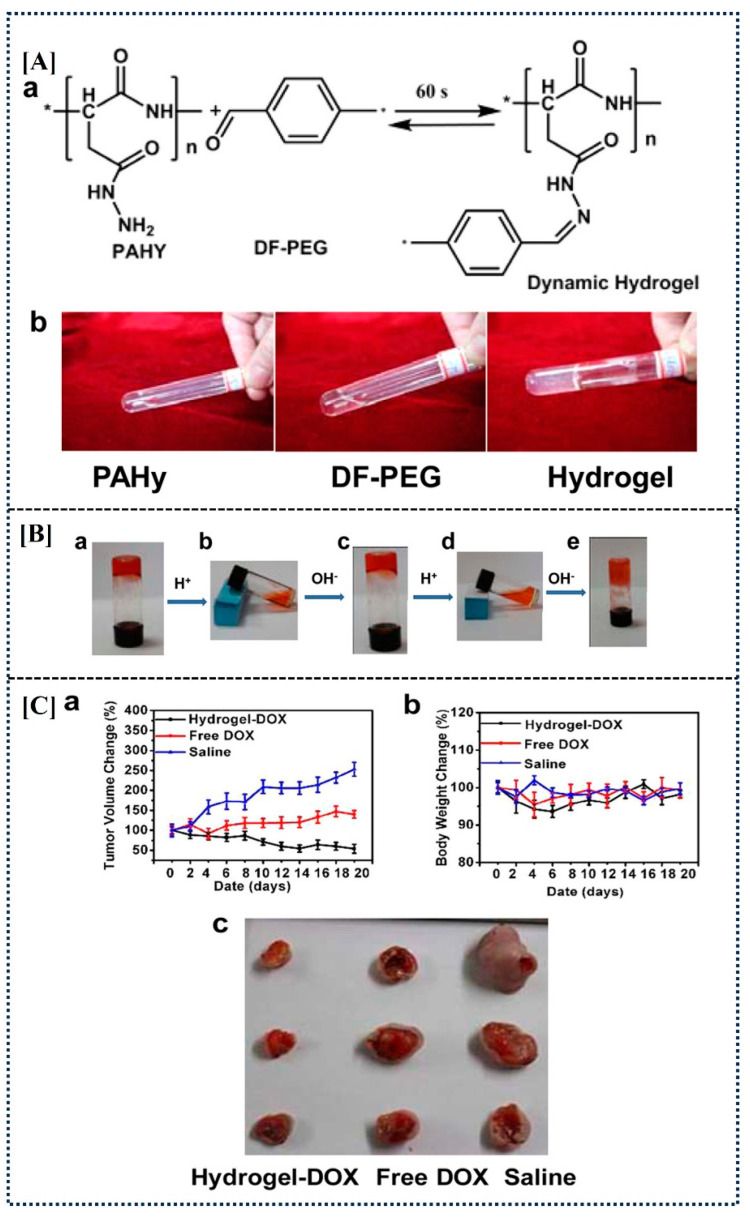
Illustration of hydrogel preparation (**Aa**). Illustration of the state of gel precursors and the hydrogel (**Ab**). Demonstration of the pH-induced phase transitions of the hydrogel: (**Ba**) gel state, (**Bb**) sol state when concentrated HCl was added, (**Bc**) recovery of the gel state when NaOH solution was added, (**Bd**) dissolution of the regenerated gel when concentrated HCl was included again, and (**Be**) the gel after five cycles of regeneration. Studies conducted in vivo following administration of free DOX and Hydrogel-DOX; time-dependent volume change curves of tumor-bearing animals (**Ca**). Tumor-bearing mice’s body weight change curves over time (**Cb**). Typical pictures of tumors removed from mice (**Cc**). Figures are reprinted from [51] with permission (Copyright © 2015, American Chemical Society).

Ortiz, J.A. et al. synthesized novel pH-responsive hydrogels by combining carboxymethylagarose (CMA) and chitosan (CS) at different weight ratios [50]. Diclofenac sodium (DS) was used as a model drug and was successfully incorporated into CMA/CS PECs. The viability of HaCaT cells was nearly 100% in the presence of the hydrogels and DS. The study demonstrated that the prepared hydrogels could be used as a pH-responsive nano system for transdermal drug delivery. Another research team synthesized pH-responsive injectable and covalently cross-linked hydrogels by mixing a dibenzaldehyde-terminated PEG (DF-PEG) solution and polyaspartylhydrazide (PAHy) solution (Figure 5A), containing doxorubicin (DOX) into the prepared hydrogel [51].

The results indicated that the sol–gel transitions of the produced hydrogel are reversible in response to pH fluctuations (Figure 5B). In an in vivo mouse model, the release rate of the DOX encapsulated within the hydrogel and its accumulation in the tumor were significantly slower compared to free DOX. The drug-loaded hydrogel exhibited enhanced efficacy, achieving approximately 80% tumor inhibition by day 20 (Figure 5C), suggesting its potential as a highly effective treatment for human fibrosarcoma with reduced side effects.

Various polymers, such as chitosan [52,53,54,55,56], poly(ethylene oxide) [57,58,59], polyethylene glycol [60,61,62], methacrylic acid [63,64,65], gelatin [66,67,68], laponite RD [69], polyethyleneimine [70], β-cyclodextrin [71,72,73], polyacrylamide, polyurethanes [74], carboxymethylagarose [50], and dibenzaldehyde-terminated PEG [51], have been used in the synthesis of pH-responsive hydrogels. Table 1 summarizes the diverse polymers that have been conjugated or encapsulated with drugs/ligands to synthesize chemical-responsive hydrogels, along with a summary of the findings.

Parisa Alipournazari et al. synthesized a pH-sensitive starch/PVA/g-C3N4 hydrogel for the delivery of DOX to breast cancer cells [80]. DOX was released faster in the tumor acidic environment compared to physiological pH conditions. Additionally, biological studies suggested that the prepared hydrogels enhanced anticancer activity and apoptosis and were non-toxic compared to free DOX. In another study, Bing Ma et al. prepared pH-responsive benzimidazole–chitosan quaternary ammonium salt (BIMIXHAC) nanogels for the delivery of DOX [81]. BIMIXHAC was cross-linked with chitosan, HA, and sodium alginates via the ion cross-linking method. Cytotoxicity studies showed that these nanogels were biocompatible and exhibited significant anticancer effects against MCF-7 and A549 cells. Zijian Zhang et al. synthesized pH- and thermo-sensitive composite hydrogels using oxidized cellulose nanofiber, PVA, and PDA via physical cross-linking [82]. In vitro studies showed that the prepared composite hydrogel exhibited a high NIR photothermal conversion efficiency, low cytotoxicity to L929 cells, pH-dependent release, and potent inhibitory effects on MCF-7 cells. Olmesartan is a hydrophobic drug that has solubility issues. To enhance its solubility and sustained release, Tahir Mahmood et al. synthesized polymeric pH-responsive nanogels developed from PEG-g-poly(MAA) using a free radical polymerization technique [63]. The resultant nanogel was able to increase the solubility by 12.3- and 13.29-fold at pH 1.2 and pH 6.8, respectively.

#### 4.1.2. Redox-Responsive Hydrogels

Redox is advantageous over other stimuli because it can change both the charge and spin states of a molecule and its assemblies at the same time. In the 18th century, Lavoisier coined the terms “oxidation” and “reduction” to describe oxygen uptake and loss, respectively [83]. Redox-active compounds offer promise in biomedical applications including drug delivery. Due to their response to redox stimuli, disulfide-cross-linked hydrogels are frequently used as a drug delivery carrier [78]. Thiols can be present in the cysteine residues of synthetic polymers as well as protein-based compounds. Therefore, thiol-based cross-linking can be used to quickly make hydrogels [84]. For the synthesis of thiol–ene and thiol–Michael cross-linked hydrogels with good biocompatibility, maleimide and divinyl sulfone (DVS) are efficient chemicals [85]. Through oxidation, thiols can also form disulfide linkages [86] and oxidants like periodate [87], hydrogen peroxide [88], and ferricyanide [89] can be used to enhance the process.

Since disulfide bonds are converted to sulfhydryl (-SH) groups in tumor tissue by high levels of reduced glutathione, most redox-responsive hydrogels are synthesized from disulfide bond-bearing compounds [42]. In normal tissue with low reducing activity, the disulfide bond is reasonably stable; nevertheless, in a tumor environment, its reduction causes structural disruptions of the drug delivery system, leading to the abrupt release of the drug [90]. Direct and indirect introduction are the two accepted methods for creating disulfide links in polymers [91]. The disulfide bond is created by oxidizing sulfhydryl groups in indirect introduction; a disulfide bond-containing moiety is inserted into the nanocarrier structure in direct introduction. The indirect method is the simpler and more popular approach among these strategies.

Kilic Boz, R. et al. used a highly effective thiol–disulfide exchange technique to develop redox-responsive hydrogels [33]. Gelation was achieved by combining linear telechelic PEG-based polymers with pyridyl disulfide units at the chain ends with thiol-terminated tetra-arm PEG polymers. Fast gelation provides macroporous hydrogels with significant water absorption through excellent conversions (>85%). Furthermore, the resultant hydrogels have the ability to self-heal because of the disulfide linkages. The hydrogels totally break down in an environment high in thiol-containing substances such as L-glutathione (GSH) and dithiothreitol (DTT). Furthermore, the molecular weight of the polymeric precursors can be changed to adjust the release profile of the encapsulated protein, in this case, bovine serum albumin. A live/dead cell viability assay confirmed the materials’ cytocompatibility. Their study suggested that redox responsive hydrogels may be appealing for a variety of biological applications due to their ease of manufacturing, and capacity to disintegrate on demand and release their payload like BSA. Other examples include DTSSP-cross-linked RZ10-RGD [79], ferric ethylenediaminetetraacetic acid (Fe-EDTA) [78], PEG-b-PBSe block copolymers [92], poly (2-methacryloyloxyethyl phosphorylcholine) (PMPC) [93], and carboxymethyl chitosan [94] polymer-based nano systems synthesized for redox-responsive drug delivery (Table 1).

Di Huang et al. prepared pH- and redox-sensitive microgels using an emulsion polymerization method [95]. These microgels were synthesized from bis(2-methacryloyloxyethyl) disulfide (DSDMA), which serves as a redox-responsive cross-linker, PEG, as the hydrophilic shell, and 2-(diisopropylamino) ethyl methacrylate (DPA) as the monomers. In vitro and in vivo studies showed the inhibition of proliferation and suppression of tumor growth. In another study, Weiyong Tao et al. synthesized a selenite-containing hydrogel composed of calcium selenite/L-arginine (L-Arg) nanospheres (SL NPs), sodium alginate, glucose oxidase, and 6-aminonicotinamide [96]. This injectable hydrogel could form a gel in a physiological environment. The prepared hydrogels efficiently induced the immunogenic cell death of tumor cells via enhanced ROS/reactive nitrogen species (RNS)-mediated combination therapy by disrupting NAPDH homeostasis and enhancing antitumor immunity. Furthermore, a TME-selective PAA-MnO_2_ hydrogel was synthesized by Akhmad Irhas Robby et al. [97]. The in vivo experiments on the cancer cell-triggered sol–gel transformation of the mineralized hydrogel showed that the PAA-MnO_2_ hydrogel can be formed in tumor-bearing mice due to its excellent ROS-scavenging activity at the tumor site, as confirmed by a gene expression analysis. Based on these reported studies, redox-responsive hydrogels could be promising in the treatment of cancer.

### 4.2. Biological Stimuli-Responsive Hydrogels

Biological stimuli-responsive hydrogels exhibit dynamic changes in their structure or properties in response to specific biological cues, enabling tailored drug delivery and tissue engineering applications [40,98]. Enzyme-responsive hydrogels, such as those triggered by proteases, enable targeted drug release at sites of inflammation or disease where elevated enzyme levels are present [99]. Glucose-responsive hydrogels, another prominent example, undergo swelling or degradation in response to variations in glucose concentration, offering precise insulin delivery for diabetes management [100]. These biological stimuli-responsive hydrogels intelligently respond to biological signals, enhancing therapeutic efficacy, and hold great promise for cancer therapy and the treatment of other medical conditions.

#### 4.2.1. Enzyme-Responsive Hydrogels

Among the several stimulus-responsive nanocarriers, the enzyme-responsive approach has gained increasing interest in the development of functional biomaterials, particularly for designing drug delivery systems, for the following reasons: (1) enzymes play important functions in most biochemical processes, and the enzyme-based method is extremely biocompatible, and (2) therapeutics can be delivered to enzyme-overexpressed tumor sites with great selectivity and efficiency using enzyme-based recognition strategies [101]. Enzyme-responsive hydrogels, leveraging specific enzyme-cleavable motifs within their polymer network, offer a dynamic platform for targeted drug delivery, tissue engineering, biosensing, and regenerative medicine [102]. These hydrogels enable spatiotemporal control over drug release, mimicking the dynamic nature of biological tissues, and providing a biomimetic matrix for promoting cell proliferation and tissue regeneration [103]. Sakai S. and Kawakami K. synthesized a novel alginate-based hydrogel incorporating phenol groups via carbodiimide coupling, aiming to develop an enzymatically cross-linkable hydrogel with gelation properties [104]. By controlling phenol incorporation, they achieved aqueous polymer solutions capable of gelation through both ionic and enzymatic cross-linking, overcoming destabilization challenges in biomedical applications. Their findings suggest the potential of phenol-modified alginate as an alternative to conventional forms for various biomedical uses.

MMPs are enzymes crucial for regulating the ECM in various physiological processes, including tissue remodeling, wound healing, and immune response modulation [105]. In cancer, MMPs play a significant role in tumor invasion and metastasis [106]. Exploiting this enzymatic activity, MMP-responsive nanocarriers, including nanoparticles and hydrogels, have emerged as promising platforms for targeted drug delivery in cancer therapy [107,108]. MMP-responsive hydrogels enable controlled drug release within tumor microenvironments, minimizing systemic toxicity. They hold promise for precision cancer therapy, enhancing efficacy while reducing adverse effects. Various MMP-based targeted hydrogels have been synthesized and explored as nanocarriers for cancer treatment [109,110,111].

Su T. et al. designed an enzyme-responsive hydrogel composed of glucose oxidase (GOx), an N-hydroxyimide–heparin conjugate, and β-D-glucose [112]. The developed enzyme-responsive hydrogel enables controlled drug release triggered by heparin-specific cleavage by heparanase, targeting cancer cells with heparanase overexpression while minimizing the negative effects of premature drug release on normal cells. Another group of researchers synthesized a matrix metalloproteinase (MMP)-responsive hydrogel to target the overexpression of O^6^-methylguanine-DNA methyltransferase (MGMT) by glioma cells [109]. They loaded O6-benzylguanine (an inhibitor for MGMT) and temozolomide (TMZ, a first-line agent for treatment) for a therapy targeting TMZ-resistant gliomas. The drug-loaded hydrogels reduced MGMT expression in vivo, rendering TMZ-resistant glioma cells more responsive to TMZ treatment. Additionally, post-surgery, these hydrogels significantly enhanced TMZ efficacy in glioma growth inhibition and reduced the recurrence of TMZ-resistant gliomas, suggesting their potential as localized medication delivery for preventing glioma recurrence. The enzymes such as proteases, hydrolases, oxidoreductases such as glucose oxidase, kinases, phosphatases, and glycosidases that are commonly utilized in enzyme-responsive hydrogels are listed in Table 2.

Other than MMPs, Hovgaard L. and Brøndsted H. synthesized dextranase-targeted enzyme-responsive hydrogels based on dextran [114]. Increasing the molecular weight of dextran, adding cross-linking agents, or lowering the amount of DMSO in the reaction mixture resulted in hydrogels with decreased swelling and higher mechanical strength. Dextran hydrogels were degraded in vitro by dextranase, as well as in vivo in rats and a human colonic fermentation model. A detailed list of enzyme-responsive hydrogels is given in Table 2. All the evidence underscores the potential of enzyme-responsive hydrogels for effective cancer treatment, offering targeted drug delivery and minimizing systemic side effects.

#### 4.2.2. Glucose-Responsive Hydrogels

Glucose-responsive systems dynamically respond to fluctuations in the surrounding glucose concentration, offering significant promise for diabetes and cancer treatments [117]. Research on glucose-responsive systems can be categorized along two dimensions: the mechanism of glucose responsiveness and its application [118]. Mechanistically, these systems fall into three main groups: lectin-based, glucose oxidase (GOx)-based, and phenylboronic acid (PBA)-modified systems. Application-wise, they are typically classified into two groups: glucose concentration diagnostics and insulin release facilitation. In major studies, glucose-responsive components such as glucose-binding proteins [119], phenylboronic acid (PBA) [115,120,121] and enzymes such as glucose oxidase/catalase (GOx/CAT)) [122,123] are used for the delivery of insulin (Table 2).

Li X. et al. designed novel, biocompatible, glucose-responsive hydrogels containing GOx, catalase, and insulin in peptide IA-0 for insulin delivery via the self-assembly approach in physiological conditions [116]. The in vitro and in vivo studies demonstrated that the developed hydrogels regulated the blood glucose levels, even in mouse models with STZ-induced diabetes. Glucose-responsive hydrogels find more extensive application in diabetic disease management compared to cancer therapy, primarily due to the higher prevalence of diabetes and the urgent need for precise insulin delivery in diabetic patients. These studies demonstrated that biological stimuli-responsive hydrogels, including enzyme- and glucose-responsive ones, are increasingly popular in the treatment of various diseases, including cancer, due to their ability to deliver drugs precisely and effectively in response to specific biological triggers.

### 4.3. Physical Stimuli-Responsive Hydrogels

To ensure the effective transport of nanocarriers within cells, it is imperative to thoroughly evaluate the appropriate designs of the drug carriers that use either active or passive targeting strategies. Common mechanisms involve receptor-mediated endocytosis, passive diffusion, or particle phagocytosis [124]. For example, the enhanced permeability and retention (EPR) effect has frequently underpinned passive targeting approaches in cancer therapy [125]. Conversely, active targeting can be achieved by incorporating specialized ligands, such as antibodies, oligonucleotides, and peptides, that bind to specific cell surface biomarkers or overexpressed receptors. Ensuring the efficient release of drugs that are conjugated, complexed, or encapsulated into the nanocarriers is a critical consideration [126]. The stability of the drug delivery system, the drug’s bioavailability, and the requisite mechanism for controlled release can all be influenced by the chemical nature of the bond and linker [127]. Recently, controlled drug release has also been achieved through the application of physical stimuli such as light, temperature, pressure, magnetic fields, and ultrasound to overcome hurdles observed in chemical stimuli-responsive hydrogels [128]. Here, we discuss thermo- and light-responsive hydrogels used for drug delivery (Table 3).

#### 4.3.1. Thermo-Responsive Hydrogels

This form of drug delivery has significantly enhanced patient convenience and maximized the efficiency of the drugs used. Nevertheless, the hypodermic route of administration has not fully addressed certain challenges, notably cardiotoxicity, that are associated with the intravenous delivery of chemotherapeutic agents such as Herceptin [142]. An alternative therapeutic approach, aiding localized continuous medication administration, has emerged as a promising strategy for enhancing therapeutic efficacy while mitigating systemic adverse effects. Thermo-responsive hydrogels undergo a reversible sol–gel transition in response to temperature changes, making them valuable for controlled drug delivery and tissue engineering [143]. For instance, injectable and thermosensitive hydrogels have been widely used for targeted drug delivery for the treatment of various diseases [144]. Below their lower critical solution temperature (LCST), they exist as a sol, becoming a gel above this threshold. This transition allows the encapsulation and targeted release of drugs, minimizing systemic side effects and improving therapeutic efficacy [145]. Common polymers used include poloxamers, poly(N-isopropylacrylamide) (PNIPAAm), CS/β-glycerophosphate, hyaluronic acid (HA), poly (ethylene glycol) (PEG), poly(phosphazene), and poly (lactic-co-glycolic acid) (PLGA), offering versatility and tunability for various biomedical applications [146,147].

**Figure 6:**
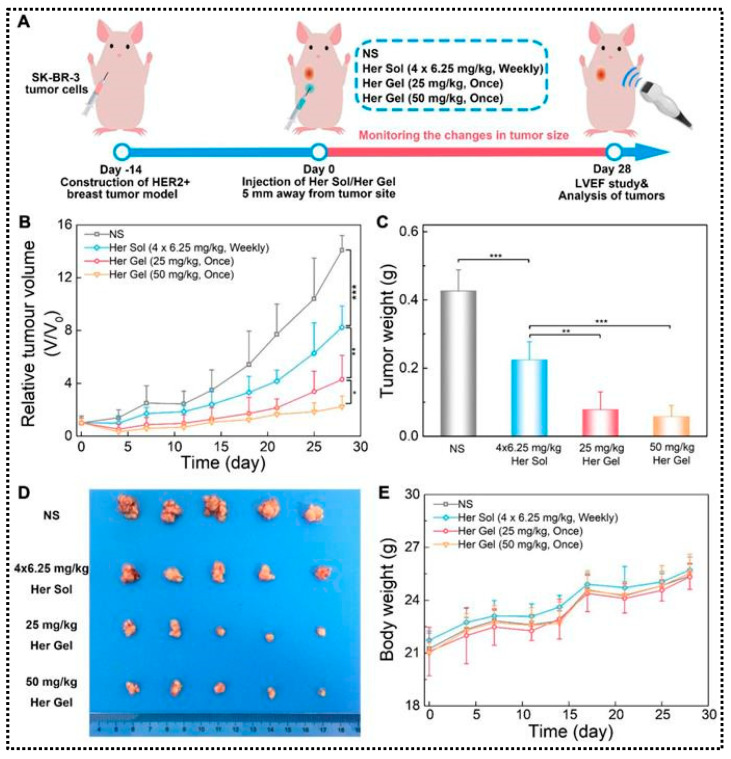
In vivo antitumor efficacy of Herceptin-loaded hydrogel. Illustration depicting the development of a HER2+ breast tumor relapse nude mice model (**A**). Tumor volume changes (**B**) and ex vivo tumor weight (**C**) on day 28 after treatment. Digital images of tumors (**D**) and body weights of mice following the various treatments (**E**). (* *p* < 0.05, ** *p* < 0.01 and *** *p* < 0.001). Figures are reprinted from [142] with permission (Ivyspring International Publisher).

In general, these materials are low-viscosity sols at low or room temperature, making it easy to entrapped anticancer therapeutic agents in polymer-, polypeptide-, and protein-based hydrogels [148]. Following injection into the body, they undergo a temperature-triggered sol–gel transition, transforming into semisolid gels. This enables the controlled release of the loaded anticancer drugs from the hydrogel in a predetermined and regulated manner [149]. Among them, thermosensitive hydrogels composed of PLGA and PEG triblock copolymers have gained popularity due to their convenient one-pot synthesis method and a good safety profile. Furthermore, both are FDA approved (PLGA and PEG) and have been used clinically for many years [150]. Chen X. et al. developed thermo-responsive injectable hydrogels composed of PLGA-PEG-PLGA triblock copolymers, with different ratios of PLGA and PEG using bulk ring-opening copolymerization [142]. The mixture of PLGA and PEG was optimized in such a way that the hydrogel exhibited sol–gel transitions in water as the temperature increased. Then, Herceptin was loaded into this designed hydrogel and then its in vitro and in vivo antitumor efficacy was assessed using a HER2+ breast cancer tumor model (Figure 6).

A single injection of the Herceptin-loaded hydrogel, combined with weekly injections of a Herceptin solution, effectively prevented tumor recurrence in a locally recurrent HER2+ breast tumor nude mouse model. Furthermore, the weekly pulsed injection of the Herceptin solution led to a significant decrease in the left ventricular ejection fraction (LVEF) in both the anticancer and anti-relapse studies. Conversely, the slow and sustained release of Herceptin from the hydrogel depot effectively mitigated cardiotoxicity. These findings suggest that the hydrogel system can enhance therapeutic efficacy, reduce systemic adverse effects, and decrease the frequency of administration in the treatment of HER2+ breast tumors. In addition to PLGA and PEG polymers, various other polymers have been utilized, such as chitosan [129], MXene nanosheets [133], L-lysine- and L-alanine-based diblock copolypeptides [131], and poly(N-isopropylacrylamide) [134,136], to synthesize thermo-responsive hydrogels. Anticancer drugs such as DOX, PTX, and CTX have been encapsulated within polymer-grafted hydrogels, which release them at predetermined times at the tumor target site. This ultimately enhances the anticancer efficacy of the agents.

#### 4.3.2. Light-Responsive Hydrogels

**Figure 7:**
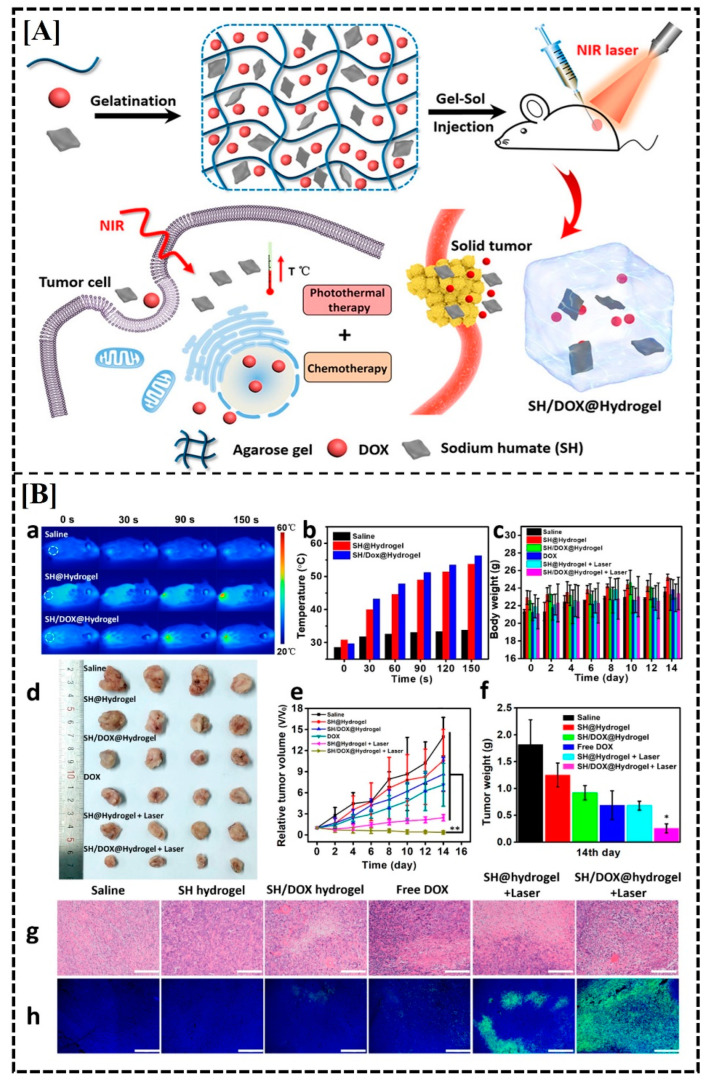
Schematic diagram of the mechanism of action of the prepared SH- and DOX-loaded light-responsive hydrogel (**A**) and in vivo anticancer activity (**B**). Thermographic imaging (**Ba**), matching temperature variation of mice under laser irradiation (**Bb**), change in mouse body weight over time (**Bc**), images of the excised tumors on the 14th day after different treatments (Bd), change in normalized tumor volume over time (**Be**), average weight of excised tumors on the 14th day after different treatments (**Bf**), histopathological analysis of excised tumors and H&E staining (**Bg**) and TUNEL staining on the 14th day after various treatments (**Bh**) (scale bars: 200 μm). Figures are reprinted from [140] with permission (Copyright © 2018 American Chemical Society).

Light-responsive hydrogels represent a cutting-edge class of biomaterials designed to undergo controlled changes in response to light [151]. These hydrogels typically incorporate light-sensitive moieties, such as photoactive molecules or photo-responsive polymers, enabling precise spatiotemporal control over their properties and functions [152]. Through techniques like photopolymerization or photoisomerization, light can trigger reversible alterations in the hydrogel’s structure, leading to phenomena such as swelling, contraction, or release of encapsulated drugs for cancer therapy [153]. This unique responsiveness to light makes these hydrogels invaluable for a wide range of applications, including drug delivery, tissue engineering, biosensing, and actuators in soft robotics, offering unprecedented levels of precision and versatility in the biomedical and technological fields [154]. Drug release mechanisms including photo-induced transformation [155], bond cleavage [156], photoisomerization [157], or photo-cross-linking [158] can be activated by UV light. Consequently, certain linkers containing aromatic rings are susceptible to UV light-induced drug release via one or more photon excitation mechanisms operating at specific wavelengths. Hou M. et al. developed a light-induced hydrogel composed of the natural product humic acid/agarose [140]. This hydrogel was utilized to encapsulate sodium humate (SH) and DOX, resulting in combined chemo- and photothermal therapeutic effects (Figure 7A). Since SH is a strong absorber of near-infrared (NIR) light, it has the ability to effectively convert light energy into thermal energy, which causes localized hyperthermia. This, in turn, causes sustained drug release from the hydrogel complex via a standard gel–sol transition, which improves the uptake of therapeutic drugs by cells. Furthermore, when solid tumors were exposed to NIR laser radiation, intratumoral injection of the drug-loaded hydrogel produced a simultaneous chemo-photothermal therapeutic response that may prevent overall tumor recurrence. In vivo studies suggested an improved antineoplastic efficacy of this prepared hydrogel compared to local delivery of free drugs to tumoral tissues (Figure 7B).

This research offers a promising start towards creating an affordable, light-responsive hydrogel for targeted tumor treatment. Chondroitin sulfate methacryloyl (CSMA)- [138] and methylcellulose [141]-like polymers are being investigated for crafting light-induced hydrogels, which could be pivotal in cancer treatment. These innovative hydrogels that are responsive to light stimuli, can adeptly encapsulate potent therapeutic agents like DOX, facilitating precise chemo- and photothermal therapy, thereby heightening efficacy in combating cancer cells [137,140,159].

### 4.4. Multi-Responsive Hydrogels

Multi-responsive hydrogels exhibit dynamic behavior in response to multiple external stimuli, such as pH, temperature, light, ions, or enzymes [160]. These versatile materials possess the ability to undergo reversible changes in their structure, properties, and functionalities upon exposure to specific physical and chemical environmental cues, allowing for the fine tuning of their behavior [98]. By incorporating multiple responsive elements into their polymer networks, these hydrogels offer enhanced adaptability and functionality, making them highly attractive for a wide range of applications in drug delivery, tissue engineering, sensing, and soft robotics [161]. Their ability to integrate and respond to various stimuli provides researchers with a powerful platform for designing sophisticated biomaterials capable of addressing complex biological and technological challenges, with potential implications for personalized medicine, regenerative therapies, and advancements in material science [162].

Multi-responsive hydrogels are comprised of three-dimensional polymer networks constructed from either natural or synthetic polymers [163]. Commonly used natural polymers include alginate, chitosan, collagen, hyaluronic acid, and gelatin. Additionally, synthetic polymers like polyethylene glycol (PEG), polyacrylamide (PAAm), and poly(N-isopropylacrylamide) (PNIPAAm) have found wide application in hydrogel synthesis (Table 4). The selection of the polymer is contingent upon the desired properties and intended applications of the hydrogel.

Liang, Y. et al. designed a pH and glucose dual-responsive multifunctional hydrogel for the specific release of metformin [121]. The hydrogel was formed using phenylboronic acid and benzaldehyde bifunctional polyethylene glycol-co-poly(glycerol sebacic acid)/dihydrocaffeic acid and l-arginine-cografted chitosan (PEGS-PBA-BA/CS-DA-LAG) (PC hydrogels) via dynamic bonds between Schiff bases and phenylboronate esters. The conductivity and hemostasis were enhanced with rGO@PDA. They evaluated tissue adhesion, blood coagulation, in vivo hemostasis, biocompatibility, antibacterial and antioxidant effects, the self-healing ability, rheological and mechanical properties, as well as in vitro metformin release. They also assessed the healing of type II diabetes foot wounds in a rat model. Their research revealed that the developed PC hydrogels were effective in accelerating the healing of chronic athletic diabetic foot wounds. These hydrogels displayed increased adhesion, stimuli-responsive metformin release, and self-healing properties.

Another study conducted by Wu Y. et al. developed a pH/ROS dual-sensitive injectable glycopeptide hydrogel by combining PBA-grafted oxidized dextran (POD) and caffeic acid-grafted ε-polylysine (CE) [167]. The hydrogel exhibited inherent antibacterial and antioxidant properties. pH-responsive micelles were used to encapsulate the angiogenesis-promoting compound mangiferin (MF). Following this, drug-loaded micelles and diclofenac sodium (DS), which is well known for its anti-inflammatory qualities, were added to the hydrogel. The results of the in vitro and in vivo experiments demonstrated the biocompatibility of the hydrogel in addition to its first-rate anti-infection, anti-oxidation, and anti-inflammatory qualities, which were subsequently followed by enhanced angiogenesis and expedited wound healing [167]. All things considered, this innovative glycopeptide hydrogel offers a straightforward and effective method for curing long-term diabetic wounds.

**Figure 8:**
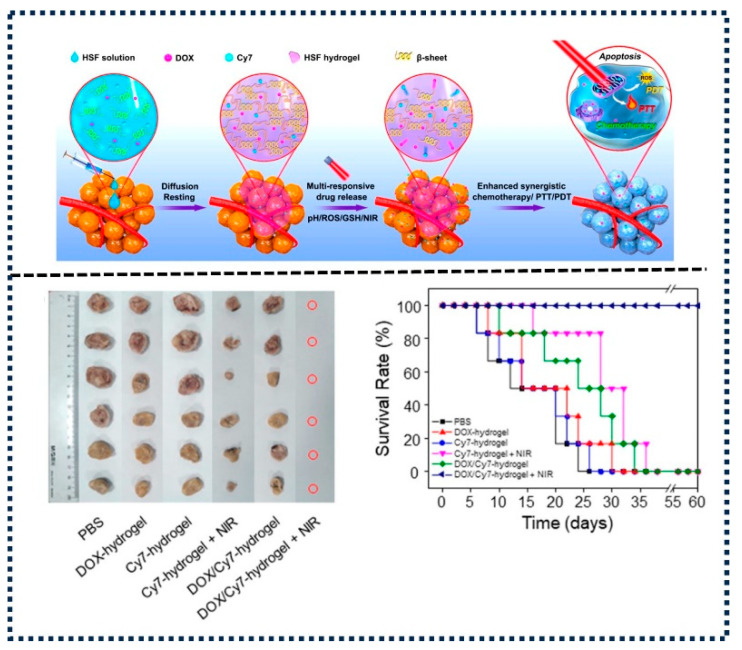
Schematic representation of multi-responsive silk fibroin-based hydrogel synthesis, action of mechanism, and in vivo antitumor efficacy. Figures reprinted from [174] with permission (Copyright © 2020, American Chemical Society).

Recent advancements in this field include the development of smart hydrogels capable of responding to dynamic changes within the TME, offering personalized and responsive treatment strategies. The available literature highlights the potential of multi-responsive hydrogels in revolutionizing cancer therapy through enhanced drug delivery and therapeutic efficacy [171,172]. Multi-stimuli-responsive hydrogels, synthesized from hyaluronic acid and a diselenide-based cross-linker, can facilitate the controlled release of DOX [173]. In vitro studies confirm the hydrogels’ cytocompatibility and demonstrated their comparable antitumor efficacy to free DOX. Moreover, DOX + ICG-loaded hydrogels display enhanced antitumor effects post-NIR irradiation [173]. Gou S. et al. also synthesized silk fibroin-based hydrogels (HSFs) for localized treatment [174]. The resulting HSF hydrogel demonstrated a clear thixotropic behavior, viscoelasticity, and self-healing capabilities. Interestingly, upon the usage of different stimuli like ROS, acidity, glutathione, heat, and NIR irradiation, this hydrogel also demonstrated good stimuli-responsive drug release characteristics (Figure 8).

This implies that in reaction to the NIR irradiation and the TME, the hydrogel could enable on-demand drug release at the desire location and time. The combination of an intratumoral injection of the prepared DOX-loaded hydrogel and NIR irradiation produced the most potent anticancer effect of all the treatment groups. This demonstrates the strong synergistic benefits of photothermal, photodynamic, and chemotherapy treatments. Interestingly, the hydrogel was able to almost completely remove the tumor masses, significantly prolonging the tumor-bearing mice’s survival time for almost 60 days without causing any negative effects (Figure 8). This underscores the potential of developing a multi-stimuli-responsive injectable HSF hydrogel as a targeted and cooperative cancer treatment platform.

**Figure 9:**
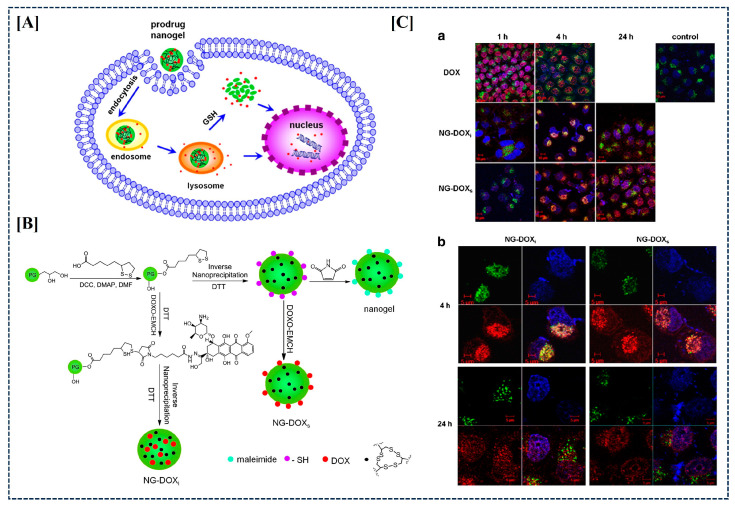
Uptake and release of DOX from nanogel (**A**). Synthesis mechanism of DOX nanogel (**B**). Cellular (intracellular/subcellular) uptake of DOX nanogel within HeLa cells (**Ca**,**Cb**). Figures reprinted from [176] with permission (Copyright © 2013 Elsevier B.V.).

Nanogels are nanoscale hydrogels, and can offer similar properties to macroscopic hydrogels but with the advantage of intravenous injection, enabling access to hard-to-reach body parts through enhanced permeability and retention (EPR). Their cellular uptake makes them ideal for safely delivering therapeutic substances, such as chemotherapeutics and nucleic acid-based medications, directly to the target cell’s cytoplasm [175]. As an example, for the delivery of DOX, Xuejiao Zhang et al. developed a pH and redox dual-responsive prodrug nanogel using an inverse nanoprecipitation technique (Figure 9A) [176]. Microscopy and flow cytometry studies on cell cultures demonstrated that the tumor cells effectively internalized the prodrug nanogels (Figure 9C).

The various nanogels and microgels that were previously discussed have shown significant potential for the encapsulation, intracellular delivery, and facilitating the enhanced anticancer efficiency of therapeutic drugs. The research findings indicate that single-, dual-, and multi-stimuli-responsive hydrogels represent a promising platform for targeted cancer treatment.

## 5. Limitations of Hydrogels

Stimulus-responsive hydrogels offer versatile platforms for drug delivery and tissue engineering, yet they are not without limitations. Hydrogels, while versatile in drug delivery, present drawbacks including a limited loading capacity, potential burst release leading to uneven drug concentrations, stability issues under varying environmental conditions, and sometimes complex fabrication processes [177]. Another significant challenge is achieving precise control over their responsiveness to external cues such as pH, temperature, or biomolecular signals, which can vary widely within biological systems [178]. This lack of fine tuning may lead to inadequate therapeutic responses or unintended side effects. Moreover, the mechanical properties of stimuli-responsive hydrogels, including their stiffness and elasticity, may not always match the requirements of the target tissue, potentially limiting their applicability in certain clinical scenarios [161]. Hydrogels like calcium alginate have low mechanical strength whereas agar/alginate hydrogel beads provide improved mechanical strength and controlled drug release [179,180].

Pre-formed hydrogels and scaffolds in tissue engineering face challenges such as infection risk and difficulties with surgical implantation. Injectable hydrogels systems offer a solution to some of these problems [181,182]. Hydrogels are often insufficient carriers for small-molecular-weight and hydrophobic drugs [183,184]. Copolymers of methyl methacrylate and acrylic acid can incorporate drug-loaded colloidal carriers to achieve homogeneous dispersion of hydrophobic drugs [185]. Introducing hydrophobic domains and using stimulated release triggers can improve controlled drug release [186]. Incorporating drug-loaded colloidal carriers into hydrogels is challenging, prompting the development of mixed delivery systems such as liposome-in-hydrogel formulations. pH-dependent hydrogels often exhibit limited solubility, and a slow sol–gel transition phase. Chemical modifications can enhance their solubility profile and mucoadhesive property [187]. Light-responsive hydrogels tend to have slow and variable responses to light stimuli. Solutions include fabricating cross-linked polymers of 2-hydroxyethyl methacrylate functionalized with azobenzene groups and forming an interpenetrating polymer network between polyacrylamide and polyacrylic acid [188,189].

Additionally, concerns regarding biocompatibility, immunogenicity, toxicity, and the potential for the long-term accumulation of degradation by-products in vivo necessitate rigorous evaluations for their safe and effective use in clinical practices [190]. The challenges found with hydrogels reveal an opportunity to enhance them by improving their responsiveness to various signals, such as temperature and light changes. This could result in the development of smarter and more sensitive hydrogels that respond better to stimuli like temperature, significantly enhancing the precision of treatment delivery and facilitating their use in medical applications.

## 6. Conclusions and Future Directions

Stimulus-responsive hydrogels represent a rapidly advancing field that is setting new benchmarks for the advancement of drug delivery platforms, offering numerous benefits over conventional therapeutics and delivery methods. As outlined in this review, the versatility of stimulus-responsive hydrogels allows for the targeted engineering for diverse biomedical applications, demonstrating a gain-of-function effect. This review presents the promising potential of stimulus-responsive hydrogels that are engineered considering biological, chemical, and physical stimuli, particularly for drug delivery in cancer therapy. While this review primarily addressed drug delivery applications of stimulus-responsive hydrogels, the ongoing research extends across multiple domains including controlled drug delivery, biocatalysis, optical sensing, imaging, cell encapsulation, and tissue engineering. In the future, synergistic therapeutic approaches (i.e., local and systemic) to regulate prolonged drug release via specialized stimulus responsive hydrogels could signify a new paradigm in cancer treatment. Future studies should also aim to further enhance the application and effectiveness of stimulus-responsive hydrogels in these diverse areas.

## Figures and Tables

**Table 1:** List of chemical stimuli-responsive hydrogels.

Stimulus	Polymer(s)	Drug/Dye/Ligand	Cross-Linking Agent	Preparation Method	Cancer/Model/Route	Findings	Reference
pH	CS and PEO	Amoxicillin, Metronidazole	Glyoxal	Cross-linking	Peptic ulcers	In the acidic environment of the stomach fluid, prepared hydrogels may be helpful for the localized administration of antibiotics.	[52]
	PAA: PEO	SAM, NAM, CHC, PDN	TDIC	Cross-linking	GI tract	The pH-dependent swelling of IPN granules in the matrix significantly determines drug release across all studied types.	[57]
	Gelatin: PEO	Riboflavin	Glyoxal	Cross-linking	For oral delivery	Gelatin and gelatin-PEO hydrogels swell based on pH and high--eight PEO.	[60]
	PEG and L-Lactide	DOX and TET	MA–PLLA–PEG–PLLA–MA	Cross-linking	GI tract	pH-mediated drug release observed (slow release of DOX in acidic buffers as well as fast release of TET).	[75]
	PEG-6000 and MAA	-	MBA	Radical polymerization reaction	Male albino rabbits	The nanogels were well-tolerated with no toxic effects in animals.	[63]
	Gelatin and Pluronic F127	CUR	FCHO	Schiff base cross-linking	Diabetes mellitus	Displayed antibacterial and antioxidative activity and biocompatibility, facilitated wound closure, and enhanced tissue regeneration.	[76]
	Lap^®^/CS/PVA	CUR	Lap^®^CUR	Cross-linking	Breast cancer cells (MDA-MB 231) and bacteria *S. aureus*, *E. coli*, and *H. pylori*	Had good blood compatibility, excellent antioxidant properties, and antibacterial activity.	[69]
	PEI-Co-MAA	Mesalazine	MBA	Free radical polymerization	Colorectal diseases	Hydrophilic drugs can be delivered to colon sites via hydrogels.	[70]
	Black seed extract and β-CD, MAA	Perindopril Erbumine	MBA	Free radical polymerization	-	At alkaline pHs, hydrogels demonstrated more swelling and in vitro drug release compared to acidic pHs, with no adverse effects observed in animals.	[71]
	CMTKG/PVP/PAM	DS	MBA	Free radical polymerization	-	A higher drug release was observed at physiological pH (pH 7.4) compared to acidic pH (pH 1.2) from hydrogel.	[77]
	PU−PEI and PU−CA	Ciprofloxacin, Bromophenol blue and Pyronin Y	-	Aminolysis and Stiglich esterification mechanism, physical cross-linking	Chronic wounds	PU–PEI films exhibited significantly higher antibacterial activity than PU–CA films, and they discharged more cargo at an acidic pH than PU–CA films did at an alkaline pH.	[74]
	CMA and CS	DS	-	Ionic complexation	Dermal drug delivery, HaCaT cells	The viability of HaCaT cells was nearly 100% in the presence of hydrogels and DS, indicating the potential of CMA/CS PECs for pH-responsive dermal drug delivery.	[50]
	DF-PEG, PAHy	DOX	-	Chemical cross-linking	Human fibrosarcoma	The DOX-loaded hydrogel exhibited enhanced efficacy, achieving approximately 80% tumor inhibition by day 20, suggesting its potential as a highly effective treatment for human fibrosarcoma.	[51]
Redox	DPA-DSDMA, PEG	PDS, BSA	-	-	L929 mouse fibroblasts	When hydrogels were treated with thiol-containing reducing agents, they broke down quickly, facilitating the release of the encapsulated payload (such as BSA) more quickly.	[33]
	PEG-SH and Fe-EDTA	Dextran	DVS	One-pot cross-linking	NIH/3T3 mouse fibroblasts	These gels offer a potentially useful platform for separating the behavior of degradation in response to reduction stimuli from the initial mechanical properties.	[78]
	Resilin	RGD	DTSSP	-	NIH/3T3 fibroblasts	Demonstrated the degradation and cytocompatibility of DTSSP-cross-linked RZ10-RGD, showcasing their potential for biomedical applications.	[79]

Abbreviations—CS: chitosan; PEO: poly (ethylene oxide); SAM: salicylamide; NAM: nicotinamide; CHC: conidine·HCl; PDN: prednisolone; PAA: poly(acrylic acid); TDIC: tolylene-2;4-diisocyanate; PLLA-PEG-PLLA: poly(L-lactide)-co-polyethyleneglycol-co-poly(L-lactide); MA: methacrylic anhydride; DOX: doxorubicin; TET: tetracycline; GI tract: gastrointestinal tract; MAA: methacrylic acid; MBA: N-methylene bis(acrylamide); CUR: curcumin; FCHO: benzaldehyde-modified Pluronic F127 polymer; Lap^®^/CS/PVA: laponite RD/chitosan/polyvinyl alcohol; PEI: polyethyleneimine; β-CD: β-cyclodextrin; CMTKG/ PVP/PAM: carboxymethyl tamarind kernel gum/polyvinylpyrrolidone/polyacrylamide; DS: diclofenac sodium; PU−PEI: polyurethane–polyethylenimine; PU−CA: polyurethane–carboxylic-acid-modified films; CMA: carboxymethylagarose; DF-PEG: dibenzaldehyde-terminated PEG; PAHy: polyaspartylhydrazide; PEG: poly(ethylene glycol); PDS: pyridyl disulfide; DPA: 2-(Diisopropylamino) ethyl methacrylate, DSDMA: Bis(2-methacryloyloxyethyl) Disulfide, BSA: bovine serum albumin; DVS: divinyl sulfone; Fe-EDTA: ferric ethylenediaminetetraacetic acid; DTSSP: 3,3′-dithiobis(sulfosuccinimidyl propionate).

**Table 2:** List of biological stimuli-responsive hydrogels.

Stimulus	Polymer(s)	Drug/Dye/Ligand	Cross-Linking Agent	Preparation Method	Cancer/Model/Route	Findings	Reference
Enzyme (HRP)	Alginate and tyramine	Phenol	HRP/H_2_O_2_	Enzymatic cross-linking	-	Findings demonstrated the viability of a unique alginate synthesized with phenols as an alternate to typical unmodified alginates.	[104]
Enzyme (GOx)	N-hydroxyimide–heparin	DOX	EDC/NHS, Gox	Radical polymerization reaction	HeLa, HepG2, and NIH-3T3 cells	Drug was released from hydrogel in an enzyme-responsive manner.	[112]
Enzyme (MMP)	Tm	TMZ, BG	-	-	C6 glioma cells, BALB/c nude male mice, orthotropic glioma model	Hydrogels reduced MGMT expression in vivo, rendering TMZ-resistant glioma cells more responsive to TMZ treatment. Additionally, post-surgery, these hydrogels significantly enhanced TMZ efficacy in glioma growth inhibition and reduced the recurrence of TMZ-resistant gliomas.	[109]
Enzyme (β-gal)	PLGA− PEG− PLGA	5-FU, LAPONITE	2-ethyl-hexanoate as a catalyst	Bulk ring-opening co-polymerization	MCF-7, female nude mice (ICR-nu/nu), PC-3 cells	Prodrug 5-FU−β-gal and nanocomposite gels were injected locally once, and the combination had long-lasting anticancer activity in vivo with no side effects.	[113]
Enzyme (MMP)	dPG	DOX	Peptide	Nano-precipitation	HeLa cells, MCTSs, primary fibroblasts	The digested multistage pNGs demonstrated enhanced diffusive transport through a dense gel matrix. pNGs facilitated the infiltration of functional chemotherapeutic medication into deeper tissue regions in tumor-like MCTSs.	[111]
Enzyme (MMP)	PEG	DOX, MIONPs, RGDS	-	Surface-initiated photopolymerization	HeLa cells, mouse fibroblast	Targeted nanocarriers internalized and efficiently carried and released DOX into the nucleus of HeLa cells within 2 h.	[110]
Enzyme (dextranases)	Dextran	-	HDI or DDI	-	SD rats, human colonic fermentation model	Dextran hydrogels were degraded in vitro by a model dextranase, as well as in vivo in rats and a human colonic fermentation model.	[114]
Glucose	PBA and glucose	PBA	AIBN as an initiator	Radical polymerization	Insulin delivery	Mechanism was not studied but could be used for insulin delivery	[115]
Glucose	IA-0 peptide	Gox, Catalase, Insulin	-	Solid-phase method	STZ-induced diabetic mice	In vitro and in vivo studies demonstrated that the developed hydrogels can regulate blood glucose levels.	[116]

Abbreviations—HRP: horseradish peroxidase; Gox: glucose oxidase; DOX: doxorubicin; EDC: 1-ethyl-3-(3-dimethylaminopropyl) carbodiimide; NHS: N-hydroxysuccinimide; TMZ: temozolomide; MMP: matrix metalloproteinase; BG: O^6^-benzylguanine; Tm: triglycerol monostearate; MGMT: O^6^-methylguanine-DNA methyltransferase; β-gal: β-galactosidase; 5-FU: 5-fluorouracil; dPG: dendritic polyglycerol; PLGA−PEG−PLGA: poly(dl-lactide-co-glycolide)-b-poly(ethylene glycol)-b-poly(dl-lactide-co-glycolide); MCTS: multicellular tumor spheroid; MIONPs: magnetic iron oxide nanoparticles; HDI: 1,6-hexamethylenediisocyanate; DDI: 1,12-dodecamethylenediisocyanate; SD rats: Sprague-Dawley rats; PBA: phenylboronic acid; AIBN: azobis (isobutyronitrile).

**Table 3:** List of physical stimuli-responsive hydrogels.

Stimulus	Polymer(s)	Drug/Dye/Ligand	Cross-Linking Agent	Preparation Method	Cancer/Model/Route	Findings	Reference
Temperature	Chitosan	DSF	-	Physical cross-linking	SMMC-7721 cells	High biocompatibility hydrogels that quickly gelled at body temperature and showed dose-dependently greater cytotoxicity compared to the free DSF solution may be given at room temperature.	[129]
	PLA-PEG-PLA	CTX and CpG-ODN	-	-	CT26 cells, CT26-bearing mice	The outcomes demonstrated that this combined approach decreases CTX toxicity while generating a cytotoxic T cell response that efficiently suppresses tumor growth, extends survival, and significantly increases the tumor cure rate.	[130]
	L-lysine and L-alanine-based diblock copolypeptide	PTX	-	-	Glioblastoma (HK308 cells)	Hydrogel loaded with paclitaxel caused less cellular inflammation, tissue damage, and reactive astrocytes than either the hydrogel or cremaphor-taxol (the usual taxol carrier). In vivo studies suggested local tumor control and improved survival.	[131]
	HPCS and F127-CHO	ICG and BSA, CaO_2_ NPs, Bi_2_S_3_	-	Schiff-base linking	L929 cells, 4 T1 cells, BALB/c nude mice	ICG@CaO_2_-BSA nanoparticles’ CaO_2_ broke down in the TME to produce Ca^2+^ and H_2_O_2_. In addition, ICG produced ROS when exposed to NIR radiation. Furthermore, when Bi2S3 nanorods and ICG were exposed to near-infrared radiation, they produced a photothermal effect that raised the temperature of tumor tissues, which helped to precisely destroy tumor cells.	[132]
	MXene nanosheets	DOX, FeCl_2_ solution, gellan gum	-	Physical cross linking	A549 and L-929 cells	MXene@GG demonstrated superior photothermal properties and precise drug release control. Additionally, cell studies confirmed MXene@GG’s high biocompatibility and the sustained anticancer efficacy of DOX.	[133]
	CS-g-PNIPAM	GO-CET/ CPT11 and shRNA	NIPAM and MAA	Free radical polymerization	U87 cells (glioblastoma), 3T3 fibroblasts, BALB/c nude mice	In vitro studies suggested cell apoptosis, reduced SLP2 protein expression, and inhibited cell migration. In vivo studies confirmed 40% tumor size compared with the untreated control group after 12 days.	[134]
	PDLLA-PEG-PDLLA	BVZ and DOX	Stannous octoate as catalyst	Ring-opening polymerization	HaCaT and HeLa cells, HeLa xenograft nude	In vitro studies showed negligible cytotoxicity on HeLa and HaCaT cells. In vivo studies suggested that hydrogel co-loaded with BVZ and DOX effectively suppressed tumors for 36 days after a single intratumoral injection, with no harm to vital organs.	[135]
	Alginate-grafted PNIPAM	DOX	EDC, NHS, MES buffer	ATRP	AT3B-1 cells	DOX was gradually released from hydrogel, and had enhanced cellular uptake, good biocompatibility, and increased efficacy in inducing cancer cell death.	[136]
Light	Azobenzene and α-CD functionalized HA	MSNs-AuNBs, DOX	NIR radiation	In situ self-assembly	HaCaT and SCC cells, MCS	Upregulation of hyaluronidase (HAase) near the tumor tissue caused hydrogel HA degradation and the release of the drug from hydrogel, which could be taken up by tumor cells and the drug is delivered to cell nuclei.	[137]
	CSMA	Gnp substrate, LAP	405 nm laser	-	MCF-7, HepG_2_, and HeLa cells, healthy and cancer patients’ blood	Study indicated that the isolation platform had acceptable biocompatibility and had isolated the selected cells successfully. This light-responsive hydrogel has potential for use in clinical applications.	[138]
	Ti_3_C_2_ MXene/cellulose	DOX	ECH	Chemical cross-linking	HepA1-6, SMMC-7721, HepG2, U-118MG and U-251 MG cells, BALB/c or C57BL/6 mice	The results showed the promise of the nanoplatform for use in cancer therapy by demonstrating that the combination of PTT and adjuvant chemotherapy delivered via this nanoplatform destroyed tumors instantly and prevented tumor relapse. Notably, DOX was released from the hydrogel and had excellent photothermal action.	[139]
	Humic acid/agarose	SH and DOX	-	Physical cross-linking	4T1 cells, 4T1 tumor-bearing BALB/c mice	In vivo studies suggested improved antineoplastic efficacy of hydrogel drugs in tumoral tissues compared to the local distribution of free drugs.	[140]
	MC	MSNs, DOX	-	-	3T3 mouse fibroblasts and Cal27 human OSCC, female BALB/c mice	Chemotherapy and phototherapy together produced a less toxic, long-lasting synergistic antitumor impact both in vitro and in vivo.	[141]

Abbreviations—DSF: disulfiram; CTX: cyclophosphamide; CpG-ODN: cytosine-phosphate-guanine oligonucleotide; PLA-PEG-PLA–poly(D, L-lactide)-poly(ethylene glycol)-poly(D, L-lactide); ICG: indocyanine green; BSA: bovine serum albumin; CaO_2_ NPs: calcium peroxide nanoparticles; Bi_2_S_3_: bismuth sulfide nanorods; HPCS: hydroxypropyl chitosan; F127-CHO: aldehyde-modified Pluronic F127; TME: tumor microenvironment; H_2_O_2_: hydrogen peroxide; ROS: reactive oxygen species; DOX: doxorubicin; GO-CET/CPT11: CS-g-PNIPAM: chitosan-g-poly(N-isopropylacrylamide); shRNA: stomatin-like protein 2 (SLP2) short hairpin RNA (shRNA); NIPAM: poly(N-isopropylacrylamide); MAA: mercaptoacetic acid; GO-CET/CPT11: irinotecan (CPT-11) to cetuximab (CET)-conjugated graphene oxide; PDLLA-PEG-PDLLA: poly (d, l-lactide)–poly (ethylene glycol)–poly (d; l-lactide); BVZ: bevacizumab; ATRP: atom transfer radical polymerization (ATRP); NHS: N-hydroxysuccinimide; MES: 2-(N-morpholino)-ethanesulfonic acid; MSNs-AuNBs: EDC: 1-ethyl-3-(dimethylaminopropyl)carbodiimide, gold nanobipyramid (AuNB)-conjugated mesoporous silica nanoparticles; azobenzene and α-CD functionalized HA: azobenzene and α-cyclodextrin-functionalized hyaluronic acid; SCC: human squamous carcinoma cells; MSC: multicellular spheroids; CSMA: chondroitin sulfate methacryloyl; Gnp substrate: gelatin nanoparticle-modified flat glass; LAP: lithium phenyl-2,4,6-trimethylbenzoylphosphinate; ECH: epichlorohydrin; PTT: photothermal performance; OSCC: oral squamous cell carcinoma; SH: sodium humate; MC: methylcellulose; MSNs: mesoporous silica nanoparticles.

**Table 4:** List of multi-stimuli-responsive hydrogels.

Stimuli	Polymer(s)	Drug/Dye/Ligand	Cross-Linking Agent	Preparation Method	Cancer/Disease Model/Route	Findings	Reference
Temperature and pH	PNVCL, Vim, PVP	5-FU	MBA	Free radical polymerization	Neoplastic cells	Hydrogels of P(NVCL-co-VIm)/PVP across various pH and temperature conditions offer promise for targeted drug delivery applications.	[164]
Temperature and pH	PNIAAm-co-IA and CS	DOX	GP	Free radical polymerization	Breast cancer (MCF-7 cells)	Lower concentrations in an acidic environment (37 °C) demonstrated faster DOX release than a neutral pH and 40 °C. The hydrogels were cytocompatible and had negligible or no cytotoxicity according to the cytotoxicity analysis.	[165]
Temperature and pH	PGA and PNH	Lysozyme	Carbodiimide	Radical polymerization, ring-opening polymerization	-	The hydrogel’s potential as a smart drug carrier was highlighted by the quicker rates of lysozyme release at pH 7.4 along with a decreased cross-linking density and PNH content. At pH 4.0, the release of lysozyme was slowed due to protonation of the PGA portion.	[166]
pH and glucose	PEGS-PBA-BA and CS-DA-LAG	rGO@ PDA and metformin	-	Double dynamic bond between Schiff bases and phenylboronate esters	Type II diabetic foot wounds	With their increased adhesion, stimuli-responsive metformin release, and self-healing properties, the PC hydrogels were shown to be effective in helping chronic athletic diabetic foot wounds to recover.	[121]
pH and ROS	POD, CE	DS and MF	Groups from POD and CE	Schiff base linkages and boronic ester bonds	Chronic diabetic wound	Results both in vitro and in vivo studies showed anti-infection, anti-oxidation, and anti-inflammatory effects at first, which were followed by enhanced angiogenesis and faster wound healing.	[167]
Temperature and pH-	PCLA	DOX-pH-GA, BA	-	Covalent cross-linking	Hepatocellular carcinoma	The in vivo investigation demonstrated the efficacious inhibition of tumor growth by the DOX-releasing hydrogel depot. These results demonstrated the pH-responsive hydrogel’s intriguing potential for localized anticancer therapy.	[168]
pH and enzyme	HEMA and MAA	5-FU	OLZ-AC	Radical copolymerization	HCT116 colon cells, rat colonic fluid	Local 5-FU release occurred at a colon location, and high 5-FU concentrations overcame cancer therapy resistance by promoting necroptosis in colon cancer cells.	[169]
Temperature and enzyme	PEG, MMP peptide	DOX, TSLs	Michael-type reaction responsible for cross linking	Thiol–maleimide reaction, chemical cross-linking	AoAF and NIH3T3 cells	Investigations into in situ drug delivery and degradation demonstrated that the TSL-gel reacts to local environmental factors such as temperature and enzymatic stimulation.	[170]

Abbreviations—5-FU: 5-fluorouracil; PNVCL: poly(N-vinylcaprolactam); Vim: 1-vinylimidazole; PVP: polyvinylpyrrolidone; PNIAAm-co-IA: poly (N-isopropylacrylamide-co-itaconic acid); MBA: N, N′-methylene bisacrylamide; DOX: doxorubicin; CS: chitosan; PNH: poly(N-isopropylacrylamide-co-2-hydroxyethyl methacrylate); GP: glycerophosphate; PGA: poly(l-glutamic acid); CS-DA-LAG: dihydrocaffeic acid and l-arginine-cografted chitosan; PEGS-PBA-BA: polyethylene glycol-co-poly(glycerol sebacic acid); POD: phenylboronic acid-grafted oxidized dextran; CE: caffeic acid-grafted ε-polylysine; rGO@PDA: polydopamine-coated reduced graphene oxide; DS: diclofenac sodium; MF: mangiferin; DOX-pH-GA: glucuronic acid-bearing doxorubicin; BA: boronic acid; PCLA: poly(ε-caprolactone-co-lactide)-b-poly(ethylene glycol)-b-poly(ε-caprolactone-co-lactide; HEMA: hydroxyethyl methacrylate; MAA: methacrylic acid; OLZ-AC: acryloyl chloride-modified olsalazine; TSLs: liposomes; AoAFs: human aortic adventitial fibroblasts.

## Data Availability

The data presented in this study are openly available in article.
